# Clinical Advances in Thyroid Disease Assessment and Management

**DOI:** 10.3390/jcm14217510

**Published:** 2025-10-23

**Authors:** Petra Petranović Ovčariček, Luca Giovanella

**Affiliations:** 1 Department of Oncology and Nuclear Medicine, University Hospital Center Sestre Milosrdnice, 10000 Zagreb, Croatia; 2 School of Medicine, University of Zagreb, 10000 Zagreb, Croatia; 3 Gruppo Ospedaliero Moncucco, Department of Nuclear Medicine, 6900 Lugano, Switzerland; luca.giovanella.md@gmail.com; 4 Department of Nuclear Medicine, University Hospital Zurich, University of Zurich, 8091 Zurich, Switzerland

Thyroid disorders affect millions worldwide, with differentiated thyroid cancer (DTC) representing the most common endocrine malignancy. Detection rates have risen substantially, largely due to the widespread use of ultrasonography (US), creating new diagnostic and therapeutic challenges in DTC management. Current clinical practice relies heavily on sonographic features of thyroid nodules and structured reporting systems such as American College of Radiology Thyroid Imaging, Reporting and Data System (ACR TI-RADS). However, the variability in outcomes among similar imaging categories underscores the need for improved assessment methods. Likewise, the quantification of treatment response and long-term outcomes, previously a barrier to individualized therapy, is being addressed through the integration of molecular and demographic predictors into clinical algorithms.

## 1. Improving Nodule Risk Assessment

Currently used risk stratification models rely predominantly on imaging characteristics, often overlooking patient-specific factors. Integrating demographic and clinical variables such as age, gender, and size of the nodule into classification systems may improve predictive precision. Recent research has shown measurable improvements in thyroid nodule risk stratification when patient-specific factors are integrated into imaging-based classification systems [1]. Age has emerged as a strong independent predictor of malignancy, with odds ratios decreasing consistently with advancing age across all ACR TI-RADS categories. In a cohort of 1128 nodules, malignancy rates varied markedly by age group, with younger patients exhibiting a higher risk even within lower ACR TI-RADS. Nodule size remains a critical determinant, with larger nodules carrying higher malignancy rates independent of sonographic score. These findings support adjusted thresholds for recommending fine needle aspiration, particularly for sonographically “indeterminate” nodules, based on patient age and gender.

## 2. Thyroid Cancer Epidemiological Changes

An analysis of 94,892 patients from the Surveillance, Epidemiology, and End Results (SEER) database (2001–2020) revealed significant epidemiological changes [2]. The proportion of patients aged ≥ 60 years increased by 4.2%, that of males increased by 2.1%, and papillary thyroid carcinoma (PTC) cases increased by 5.3% in the 2011–2020 cohort compared with 2001–2010. Regional disease presentation rose by 3.7%, reflecting potential changes in both detection practices and disease biology. Although survival improved by 8% in the later cohort, this difference is not statistically significant. Key prognostic factors included stage, age, gender, and histopathological subtype. Patients with the follicular variant of PTC demonstrated the most favourable outcome (HR: 0.78), while those with anaplastic tumors and thyroid sarcomas showed the poorest prognosis (HR: 9.61), as expected.

## 3. Radioiodine Therapy: Modulating Clinical Indications

Radioiodine therapy remains a cornerstone in thyroid disease management. In benign conditions, its therapeutic scope is still expanding. The authors of a recent study involving patients with large toxic multinodular goiter treated with 20 mCi (740 MBq) of ^131^I reported a mean thyroid volume reduction of 36% at six months, accompanied by a 12.8% increase in tracheal diameter [3]. These findings support the use of ^131^I as an effective alternative to surgery, even in managing large goiters.

In DTC, a risk-adapted approach to ^131^I therapy has become standard practice. Treatment intent, whether ablative, adjuvant, or therapeutic, is now guided by individualized risk assessment. Post-therapy ^131^I scintigraphy provides essential staging information, underscoring the theranostic role of ^131^I [4]. Personalized therapy selection approaches consider both stimulation methods and administered activity, optimized according to patient-specific parameters.

## 4. Advances in Biomarker Development and Validation

Analytical challenges in thyroid biomarker measurement have limited the wide clinical adoption of promising markers. New-generation calcitonin (CT) immunoassays have addressed long-standing issues related to pre-analytical degradation and inter-assay variability [5,6,7]. Procalcitonin has emerged as a robust alternative or complementary marker to CT, particularly valuable when CT levels are indeterminate [8].

Evaluation of biomarker kinetics provides additional insights: CT and carcinoembryonic antigen (CEA) doubling times shorter than six months indicate poor prognosis, whereas values exceeding two years are associated with favourable outcomes. Novel biomarkers, such as pro-gastrin-releasing peptide (ProGRP) and CA19-9, are under investigation for monitoring advanced disease and assessing therapeutic response [9].

## 5. Autoimmune Thyroid Disease and Pregnancy

Recent clinical studies have clarified optimal timing for conception after ^131^I therapy in women with Graves’ disease. TRAb levels may remain elevated for up to two years post-treatment, with the risk of hyperthyroidism declining over time: 8.8% when conception occurs within 6–12 months, 5.5% at 12–18 months, and 3.6% for 18–24 months [10].

Third-trimester TRAb levels above 10 U/L are the principal risk factor for neonatal hyperthyroidism. Regarding anti-thyroid drugs, propylthiouracil increases fetal malformation risk by approximately 1.1–1.6% above baseline, while thiamazole increases risk by 2–3% depending on dose. These findings inform preconception counseling and individualize management during pregnancy [11].

## 6. Redifferentiation Therapy in Radioiodine-Refractory DTC

Pharmacological redifferentiation therapies are redefining the management of radioiodine-refractory DTC. These strategies primarily target the MAPK signaling pathway to restore sodium iodide symporter expression, thereby re-establishing radioiodine avidity [12]. Clinical trials involving the use of MEK and BRAF inhibitors have demonstrated renewed radioiodine uptake in previously refractory tumors, offering a potential shift from palliative to disease-modifying treatment [13,14,15,16,17,18,19,20,21].

## 7. Management of Rare Thyroid Disorders

Resistance to thyroid hormone (RTH) remains a rare but clinically significant condition, often complicating perioperative care. Mutations in thyroid hormone receptor genes (THRα or THRβ) result in elevated thyroid hormone levels with non-suppressed TSH and a mixed clinical phenotype with features of either hypothyroidism or hyperthyroidism. A recent case report described successful perioperative management in a pediatric patient with complex congenital heart disease and RTH [22]. Thiamazole administration selectively mitigated thyroid hormone effects on α-receptors, enabling safe cardiac surgery. This case exemplifies the importance of individualized strategies for managing rare endocrine disorders with complex comorbidities.

## 8. Knowledge Gaps and Future Research Directions

The authors of future studies should integrate molecular and genomic profiling into clinical decision-making algorithms. Validation of genetic and epigenetic predictors in large, diverse cohorts will improve risk stratification and identify novel therapeutic targets.

Artificial intelligence and machine learning offer substantial promise for enhancing diagnostic accuracy. Multimodal data integration, including imaging, molecular, and clinical features, could strengthen risk assessment and treatment selection. In addition, prospective validation of AI tools is an important research priority.

Long-term, population-based follow-up studies are essential to confirm the clinical utility of new diagnostic and therapeutic strategies. Establishing biobanks and standardized outcome registries will facilitate this progress. Finally, cost-effectiveness analyses are needed to guide the adoption of novel technologies.

## 9. Conclusions

Recent advances in thyroid medicine have led to measurable improvements in diagnosis, risk assessment, and treatment outcomes. The integration of patient-specific parameters with imaging-based classifications enhances thyroid nodule characterization. Biomarker development continues to refine both diagnostic precision and disease monitoring.

Epidemiological data reveal evolving disease patterns, while innovation in radioiodine and redifferentiation therapy expands therapeutic possibilities. Despite this progress, however, challenges persist in standardizing protocols, validating biomarkers, and achieving a true personalization of treatment.

The studies presented in this Special Issue provide evidence-based insights into the evolving domains. Continued collaborations among researchers, clinicians, and healthcare systems will be essential to translate these advances into routine clinical practice and to achieve the ultimate goal: improved patient outcomes through precision-based thyroid care.

## Data Availability

Not applicable.
